# A coevolution-guided model for the rotor of the bacterial flagellar motor

**DOI:** 10.1038/s41598-018-30293-0

**Published:** 2018-08-06

**Authors:** Shahid Khan, Tai Wei Guo, Saurav Misra

**Affiliations:** 1 Laboratory of Cell Biology, Center for Cancer Research, National Cancer Institute, NIH, Bethesda, MD 20892 USA; 2 Molecular Biology Consortium, Lawrence Berkeley National Laboratory, Berkeley, CA 94720 USA; 3 Present Address: Department of Biochemistry & Molecular Biophysics, Kansas State University, Manhattan, KS 66506 USA

## Abstract

The *Salmonella typhimurium* trans-membrane FliF MS ring templates assembly of the rotary bacterial flagellar motor, which also contains a cytoplasmic C-ring. A full-frame fusion of FliF with the rotor protein FliG assembles rings in non-motile expression hosts. 3D electron microscopy reconstructions of these FliFFliG rings show three high electron-density sub-volumes. 3D-classification revealed heterogeneity of the assigned cytoplasmic volume consistent with FliG lability. We used residue coevolution to construct homodimer building blocks for ring assembly, with X-ray crystal structures from other species and injectisome analogs. The coevolution signal validates folds and, importantly, indicates strong homodimer contacts for three ring building motifs (RBMs), initially identified in injectisome structures. It also indicates that the cofolded domains of the FliG N-terminal domain (FliG_N) with embedded α-helical FliF carboxy-terminal tail homo-oligomerize. The FliG middle and C-terminal domains (FliG_MC) have a weak signal for homo-dimerization but have coevolved to conserve their stacking contact. The homodimers and their ring models fit well into the 3D reconstruction. We hypothesize that a stable FliF periplasmic hub provides a platform for FliG ring self-assembly, but the FliG_MC ring has only limited stability without the C-ring. We also present a mechanical model for torque transmission in the FliFFliG ring.

## Introduction

Self-assembly of sub-complexes from defined protein components is a principal biochemical strategy for elucidation of the design principles of macromolecular assemblies^1–5^. The rotor ring of bacterial flagella is a remarkable assembly that couples mechanical rigidity with the capacity for rapid, long-range conformational changes. Elucidation of its architecture and structural dynamics will provide fundamental insights into the construction of energy and signal transducing ring assemblies^6^. Construction of the bacterial flagellum begins with the assembly of a transmembrane MS ring scaffold, followed by formation of the rotor module of the flagellar motor. The MS ring, which consists of multiple copies of the FliF protein, templates assembly of the rest of the flagellar base. Multiple copies of the cytosolic flagellar protein FliG attach to the cytoplasmic face of this ring to form the rotor track. The membrane stator Mot complexes step along FliG, coupling ion flux to torque generation. The cytosolic C-ring, formed by assembly of the proteins FliM and FliN onto FliG, binds CheY signal proteins to switch rotation direction with high cooperativity. Thus, the MS ring is a torque converter as well as assembly template, transmitting torque to rotate the many-micrometer-long external flagellar filament at high speed (>1000 Hz in aqueous media).

In this study, we combine residue coevolution with electron microscopy of biochemically-defined complexes to learn how the FliF ring is built and how it templates assembly of the FliG rotor ring. In structural biology, residue coevolution methods draw on information theory^7^ to score the covariance between distant residue positions in a multiple sequence alignment (MSA). MSAs have been analyzed for a long time for identification of functionally important conserved residues and for construction of homology models^8^. The use of MSAs for residue coevolution maps structural and functional interactions between both conserved and non-conserved residue positions important to the generic design of protein families^9,10^, not possible by analysis of residue conservation alone. Identification of coevolved 3D residue contacts for protein fold and protein-protein interactions is most straightforward and well-established; as documented by recent advances that exploited recent algorithm developments (e.g.^11^) to successfully predict novel folds^12^ and subunit interactions in large, multi-protein complexes^13^. Residue coevolution reveals common design principles applicable to all species in the MSA.

Cryoelectron tomography has revealed the diversity among bacterial flagellar motors^14,15^ to provide important clues regarding the mechanical design for torque generation^16^. Here, we study rotor assembly in *Salmonella enterica serovar Typhimurium* (“*Salmonella*”) based on the overproduction of biochemically defined ring assemblies that form upon over-expression of motor protein components in non-motile hosts^17–22^. The discovery of a motile, but chemotactically defective, FliFFliG fusion mutant^23^ prompted electron microscopic imaging of FliF and FliFFliG rings overproduced in non-motile *E*. *coli* expression hosts. The resulting electron microscopy (EM) data led to the first 3D reconstructions of basal flagellar assemblies^20^. However, volume differences between electron density maps of overproduced FliF and FliFFliG rings were incompatible with the molecular mass and expected stoichiometry of FliG. In contrast, density could be assigned to FliG in cylindrically-averaged reconstructions of intact basal bodies isolated from the FliFFliG fusion mutant strain^24^. The likely explanation for the difference was that the FliG component of the fusion did not form ordered rings^20^ due to the absence of other basal flagellar proteins rather than the nature of the fusion.

Since 2004, FliG crystal structures from bacteria other than *Salmonella* have revealed multiple conformations which indicate that FliG is flexible^25–28^. The structure of full-length FliG from *A*. *aeolicus* shows three primarily helical domains, FliG_N, FliG_M and FliG_C^28^. NMR and X-ray crystallographic characterization of the *T*. *maritima* or *H*. *pylori* FliG_N domains show that FliG_N alters conformation to co-fold with a C-terminal segment of FliF (FliF_C-tail_); the resulting complex adopts a conformation similar to that of FliG_M^29,30^. Type III injectisome proteins form membrane scaffolds with morphologies similar to the MS ring^31^. Inter-subunit contacts present in crystallized rings of a SctJ family member^32^ and in cryo-EM reconstructions of the *Salmonella* T3S injectisome^33^ reveal ring building motifs (RBMs) which form chained rings to assemble stable scaffolds^32,34,35^ and may also exist in flagellar MS rings^36^.

While no structures are yet available for *Salmonella* FliG or FliF, residue coevolution methods allow us to relate atomic structures of flagellar proteins from other species and non-flagellar proteins to EM maps reconstructed from overproduced FliFFliG assemblies. The current genome database has the sequence depth for identification of coevolved contacts for fold identification as well as hetero- and homo-oligomeric protein contacts. In addition, heterogeneity can be evaluated by recent 3D EM classification methods for single particle reconstructions^37^. We have integrated these developments to better understand the FliFFliG structure in an independently derived reconstruction, largely in agreement with the published map^20^, that we also present in this report. We matched residue coevolution matrics to contact maps for available crystal structures to determine generic folds and exploited outlying coevolved pairs to build dimer models with rigid-body and flexible-fit protocols. Rings generated from the dimer models of the proteins from other species or injectisome analogs accounted for the high-density volumes in our EM reconstruction. This convergence of the sequence, X-ray crystallographic and EM data identifies key design features among species that give insight into rotor assembly and allow us to propose a model for transmission of torque through the MS-ring.

## Results and Discussion

### Local resolution measures show three high-density rings in the 3D EM reconstruction

The cloning, expression, purification and negative-stain grid preparation for our structure are outlined (Methods, Supplementary Information Fig. S1A). The FliFFliG fusion protein expressed at levels comparable to those of FliF and FliG expressed separately. The overproduced FliFFliG assemblies packed the inner membrane without triggering lysis or growth arrest implying they are impermeable. Stain filled the central core of the negatively-stained isolated assemblies, matching the central orifice seen in images of freeze-etched cell envelopes.

Structural heterogeneity, which can be evaluated by 3D classification of the assemblies, as well as differences in local signal-to-noise levels influence the final reconstruction. We began with a rotationally-symmetrized reference of the *Salmonella* basal body^38^ truncated to volumes assigned for FliF and FliG^24^, followed by 3D-reconstructions generated by iterative refinement cycles successively used as references. The complexes preferentially adopted an orientation close to the en-face view, but other orientations were also sampled. Different projections are seen in the 2D class averages. The volume of the cytoplasmic module was variable as appreciated by examination of the five stable 3D class averages. The final 3D reconstruction was obtained after 3D classification and refinement of the combined particle population of two 3D classes with the more extensive cytoplasmic volume. The local resolution of its major voxel fraction was 18.4 angstroms (Methods, Supplementary Information Fig. S1B–D).

**Figure 1:**
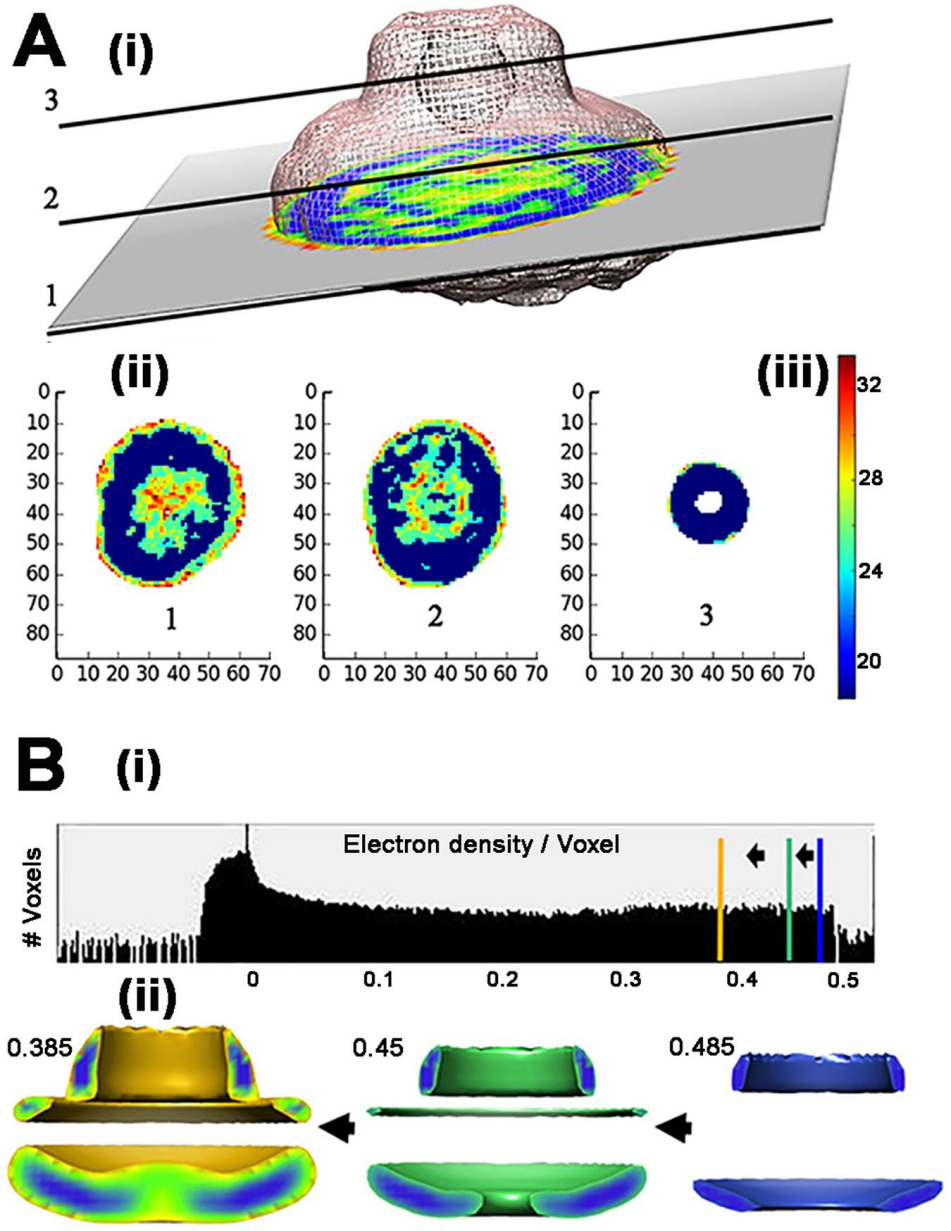
High-density sub-volumes in the 3D EM reconstruction. (**A**) ResMap^59^ was used to compute local resolution. (i) 2D slices taken at three positions (1,2,3) in the 3D-reconstruction with color-coded estimated local resolution. (ii) Local resolution maps of the three slices. Distance in nanometers. (iii) Vertical bar shows color scale for the local resolution (in angstroms). (**B**) (i) Density distribution of the rotationally averaged reconstruction^77^. Vertical bars show electron density thresholds used for the half-maps (blue = 0.475, green = 0.45, gold = 0.385). (ii) Changes in the rotationally-averaged map as threshold is decreased from 0.475 (volume = 0.76*(10^6) cubic angstroms) to 0.45 (volume = 2.14*(10^6) cubic angstroms) to 0.385 (volume = 5.93*(10^6) cubic angstroms).

No symmetry was imposed for the 3D reconstruction, but it was cylindrically averaged for detection of high-density sectors. A cylindrically-averaged reference from projection-matching of side-on images and 26-fold symmetry based on rotational power spectra of en face images was used for the previously published map^20^. The difference in image processing procedures is apparently not consequential as the maps are remarkably similar. The cylindrically-averaged map revealed three clearly-demarcated high-density volumes (Fig. 1B). We will assume that these volumes represent rigid-body sectors in the assembly. Two closely apposed zones have morphology consistent with the S-ring and adjacent top collar, based on direct comparison with the basal body reconstruction^38^. The module on the other side of the S-ring is separated from it by a distance greater than the cytoplasmic membrane. We therefore designated it as the cytoplasmic module. The heterogeneity in the cytoplasmic module revealed by the 3D class averages is probably due to the coexistence of populations with ordered and disordered FliG rings, consistent with the hypothesis of Suzuki *et al*.^20^.

### Coevolution-guided dimer models of FliF and FliG

#### FliF

**Figure 2:**
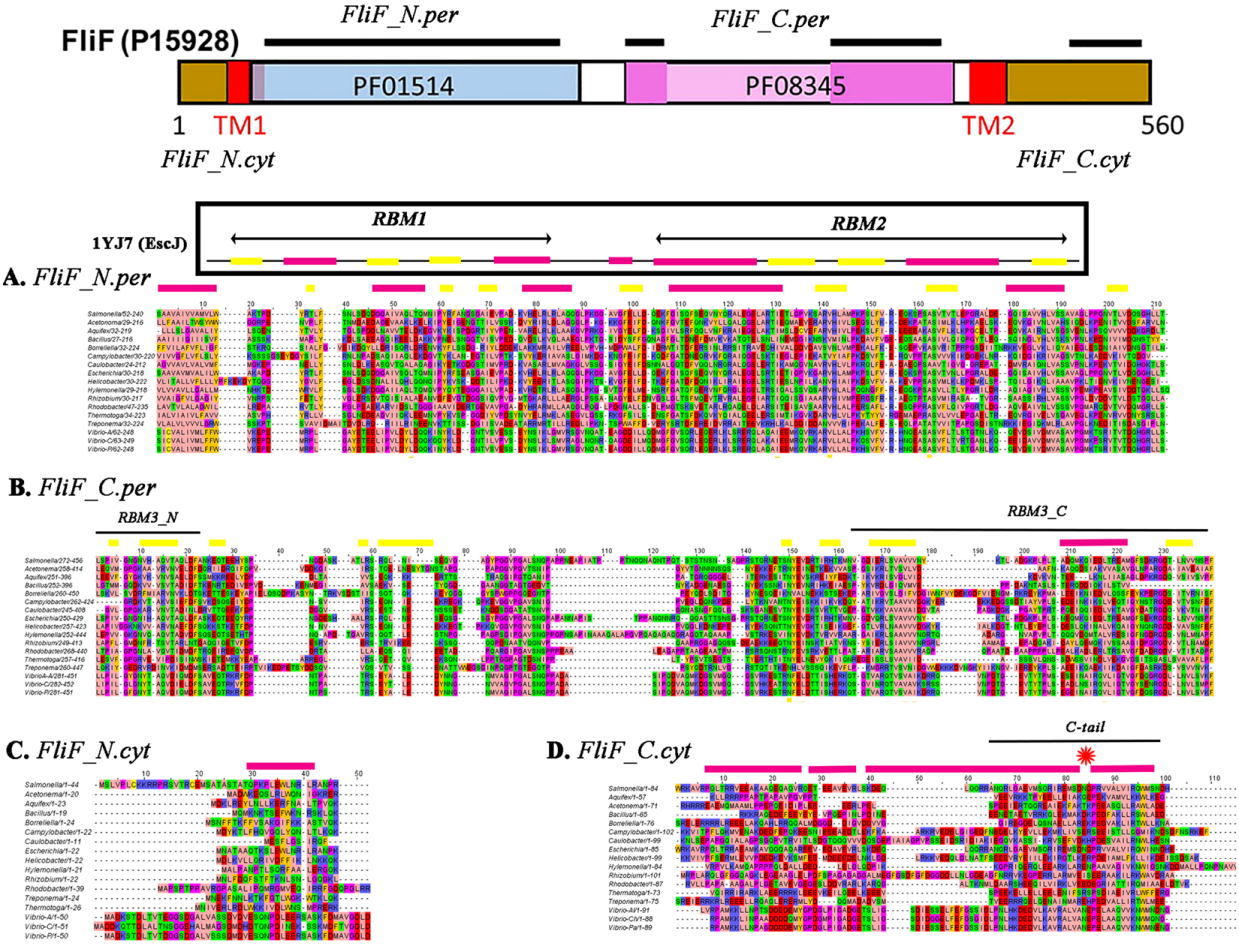
FliF domains - MSAs and secondary structure prediction. Purple bars = α-helix; yellow bars = β sheet. MSA = Multiple sequence alignment (residue coloring (Zappo)). (**A**) *Salmonella* FliF sequence (Uniprot ID P15928) with TMHMM^69^-predicted transmembrane helices (TM) and Pfam MSAs of FliF periplasmic domains. We assigned sequence segments for it and representative species to the periplasmic and cytoplasmic N- or C-terminal domains based on the boundaries of the TM helices. PsiPred predicted secondary structures were categorized into α-helix, β-sheet or neither (disordered). Pfam 01514 incorporates *Salmonella* FliF (Uniprot ID P15928) residues 33–221. Pfam 08345 incorporates Salmonella FliF residues 253–437. (**B**) MSA of the periplasmic FliF N-terminal domain (FliF_N-per) from representative species (MSA_RS_) aligned to *Salmonella* FliF_272–456_. (**C**) MSA of the periplasmic FliF C-terminal domain (FliF_C.per) from representative species (MSA_RS_) aligned to *Salmonella* FliF_272–456_. (**D**) MSA_RS_ of the cytoplasmic FliF N-terminal segment (FliF_N.cyt) aligned to *Salmonella* FliF_1–44_. (**E)** MSA of the cytoplasmic FliF C-terminal segment (FliF_C.cyt) aligned to *Salmonella* FliF_470–554_. Asterisk (red) marks hinge between α-helical segments seen in *T*. *maritima* FliF_C-tail_ crystal structure (5TDY.pdb).

Multiple sequence alignments (MSAs) with the *Salmonella* sequences as reference were constructed. Eubacterial FliF proteins have two trans-membrane helices as an ubiquitous feature. Their N-terminal and C-terminal tails are in the cytoplasm, with a large middle domain in the periplasm. We expanded a representative species set based on diversity and research focus, shown to be well distributed over the FliG C-terminal domain (FliG-C) phylogenetic tree^39^, to include species with basal structures resolved by cryo-tomography^40^. (Fig. 2).

The FliF protein was analyzed as four distinct segments; cytoplasmic N-terminal (FliF_N.cyt), periplasmic N-terminal (FliF_N.per), periplasmic C-terminal (FliF_C.per) and cytoplasmic FliF C-terminal (FliF_C.cyt). The predicted (FliF_N.per) secondary structure elements followed a pattern seen in crystals of rings (PDB ID 1YJ7) formed by the injectisome SctJ family member, EscJ^32^. The sole discrepancy between the two is in the disordered segment between RBM1 and RBM2 sequences, where a β strand was predicted for FliF but an α-helix is present in the EscJ crystal structure. The predicted (FliF_C.per) secondary structure elements were in agreement with secondary structure assignments for a split RBM model (“RBM3”)^36^. However, additional β-sheet elements were predicted in segments of the extensive disordered sequence located between the halves of the split RBM3 domain. The entire FliF_C.cyt segment was predicted to be a-helical with three gaps. One of the gaps corresponded to the hinge seen in the structure of the *T*. *maritima* FliF C-terminal 36 residues (FliF_C-tail_) in complex with FliG_N (PDB ID 5TDY). In FliF_N.cyt, a ten-residue segment conserved in all representative species was weakly predicted to be α-helical. Its significance is presently unclear.

**Figure 3:**
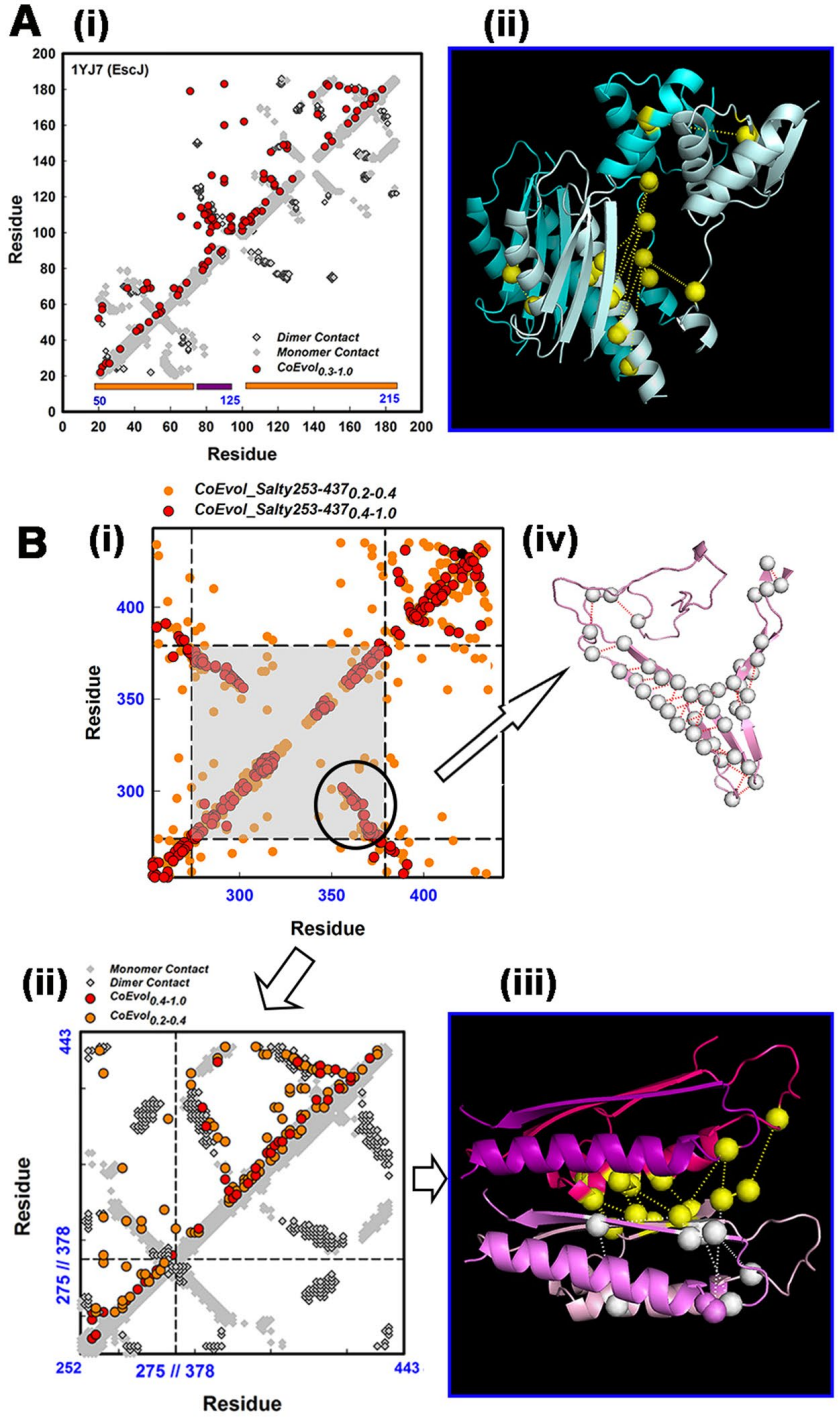
Coevolved fold and subunit contacts in the periplasmic MS-ring. (**A**) (i) FliF coevolution matrix (red) superimposed on the contact map (monomer contacts (white), dimer contacts (gray)) in the EscJ ring crystal structure (PDB ID 1YJ7). EscJ residue numbers (black); *Salmonella* residue numbers (blue). RBM motifs (orange bars), connector (purple bar) (ii) Two adjacent subunits from the 1YJ7 ring (chain 1 (blue), chain 2 (cyan)). Dotted lines link co-evolved pairs (yellow spheres) at the interface. **B**. (i) Coevolution matrix (top contacts (1.0–0.4 (red), 0.4–0.2 (orange)) for the Pfam PF08345 MSA. There is a strong coevolution signal in the central segment (gray) that separates end sequences used to construct a split RBM (RBM3) based on structural homology with RBM1, RBM2. Circle marks coevolution signal for β-hairpin. (ii) Truncated PF08345 coevolution matrix (strong (0.4–1.0 (red))/weak (0.2–0.4 (orange)) couplings) superimposed on the contact map for the split RBM3 model. Symbols as in (Ai). Dimer contacts are from our RBM3 dimer model (Methods). (iii) RBM3 dimer. (FliF_252–273_ (pink/magenta) + FliF_378–443_ (light pink/salmon)) The coevolved pairs connect the separate sequence segments in the RBM3 fold (white spheres) and adjacent RBM3 structures (yellow spheres). (iv) Coevolved couplings (circled in (i)) mapped onto a homology model of the SpoIIIAG β_2–8_ triangle motif^41^. Scores = Subscripts in symbol labels

Residue coevolution matrices were determined separately for FliF_N.per and FliF_C.per. We clustered the Pfam MSAs of the FliF super-family periplasmic N- and C-terminal segments to filter out sequences of non-flagellar homologs. We then trimmed the filtered Pfam periplasmic FliF N-terminal (FliF_N.per) MSA to remove residue positions not present in the EscJ (1YJ7) structure sequence. The top couplings of the coevolution matrix generated from this final MSA were then mapped onto the 1YJ7 subunit contact matrix (Fig. 3Ai). Contact pairs with Cα-Cα distance <10 angstroms are shown in the map, with inter-subunit contacts distinguished from subunit fold by grayscale. The mapped top coevolved residue pairs had a mean Cα-Cα distance of 15.5 ± 1.8 (sem) angstroms indicating that most participated in or were adjacent to contacts, as appreciated by superposition of the coevolved pairs onto the 3D structure (Fig. 3Aii). These top couplings constituted 1% (>2.5σ) of the coevolution matrix, with a normalized PSICOV score (Methods) of 0.3. The top couplings typically represented 1–0.2% (2.5–3σ) of the coevolution matrices analyzed in this study. The match of the off-diagonal elements between the contact and coevolution matrices provided coevolutionary information for validation of the monomer fold and the known dimer contacts mediated by the loops between the two RBMs. Henceforth, we refer to the match of the off-diagonal couplings as the “coevolution signal”. The loops, which mediate subunit contacts in SctJ injectisome rings^32,33^, are particularly rich in coevolutionary information. We conclude that the RBM1-RBM2 design is conserved and used for chained ring formation in FliF periplasmic ring assemblies.

Structural prediction from residue coevolution is more challenging for the FliF periplasmic C-terminal domain (FliF_C.per). The cryo-EM injectisome structure^33^ and a Rosetta-based structural homology model for RBM3^36^ are available. The most striking coevolution signal was not part of the model, but resided in the region (residues 274–376) reported as “disordered”^36^, between the split RBM sequence segments (circled in Fig. 3Bi). The coevolution matrix importantly though, validated the fold adopted by the separated RBM3 sequence modules and supported their association as predicted from the structural homology model^36^. The Cα-Cα distance of the coevolved residue pairs split between the modules was 9.0 ± 2.0 angstroms. Encouraged, we utilized the residual couplings to construct a dimer model. The generated model (Fig. 3Bii,iii) required minimal deformation of the RBM3 fold (root mean square deviation, RMSD = 0.35 angstrom), with close contact between the RBM3 monomers (8.1 ± 1.8 angstrom Cα-Cα distance) consistent with a rigid-body fit. The signal in the disordered region localized to sequences predicted to have β-sheet structures (Fig. 2B). A protein threading model of the region (Fig. 3Biv) shows that the couplings map to a β-hairpin, as in the recently reported triangle motif of the *Bacillus subtilis* SpoIIIAG channel^41^. There was weak sequence homology between Salmonella FliF and *B*. *subtilis* SpoIIIAG for FliF residues 274–300 (score = 27) and 335–368 (score = 30). We did not model possible contacts between this predicted structure and the split RBM3 as the coevolution signal for their association was too sparse. In conclusion, residue coevolution validated the RBM3 model and predicted the homodimer contacts required for its ring formation. In addition, there is a strong coevolution signal for an additional β-sheet structural module.

#### FliG

**Figure 4:**
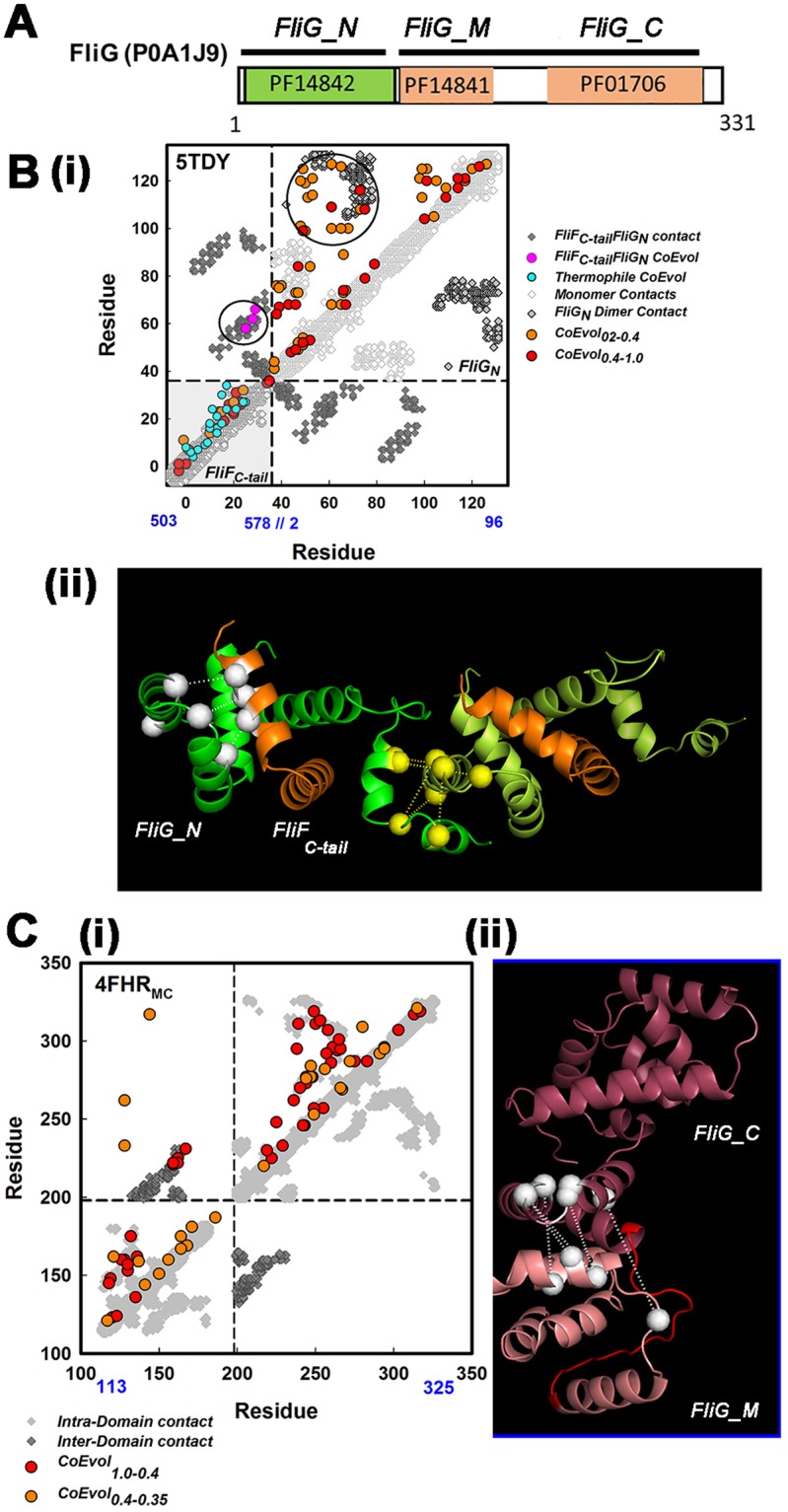
Residue coevolution supports a composite FliG_N ring and stacked FliG_MC ring. (**A**) The Salmonella FliG sequence (Uniprot ID POA1J9) with the three Pfam MSAs used for the coevolution analysis. (**B**) (i) Coevolution matrix mapped onto the contact map for the *Thermatoga* FliF_C-tail_. (gray) FliG-N complex. For the FliF_C-tail_ coevolution matrix, MSAs were seeded from *Salmonella* (red/orange) or *T*. *maritima* (cyan) sequences with HMMER3^67^. Circles denote inter-domain couplings superimposed on contacts between FliG-N (yellow) and between FliG-N and FliF_C-tail_ (olive). (ii) Interfacial FliF-FliG (white) and FliG-N dimer (yellow) coevolved pairs are shown on the dimer model of the 5TDY complex. FliG_N (green); FliF_C-tail_ (gold). Model construction was initiated as outlined for FliF RBM3, but with the FlIG_N C-terminal helix specified as fully flexible in Haddock due to its variation in crystal structures^28–30^. (**C**) (i) Coevolution matrix superimposed on the contact map for the FliG_MC crystal structure (PDB ID 4FHR). Inter-domain stacking contacts (yellow). (ii) Coevolved couplings for the stacking contact mapped onto the 4FHR structure. FliG_M_ (salmon) and FliG_C_ (mauve). Linker helix_MC_ (red). Scores for coevolved pairs noted as in Fig. 3.

We downloaded the Pfam sequences for the individual FliG domains (Fig. 4A). We focused effort on coevolution analysis of the FliG N-terminal (FliG_N) domain, which is known to adopt multiple folds due to conformational changes triggered by association with FliF_C-tail_^29,30,42^. We started with analysis of the FliF_C-tail_. We constructed separate MSAs (n > 800) from *S*. *typhimurium* and *T*.*maritima* sequences as seeds (Fig. 4B**)** to evaluate the secondary structure prediction for FliF_C.cyt. The coevolution signal indicated contiguous α-helical structure in line with the prediction. Off-diagonal elements were mostly absent, indicating lack of strong inter-helix interactions. The coevolution signal was concentrated in the last twenty C-terminal residues of the FliF_C-tail_ α helix and matched the contact map for its structure in the *T*. *maritima* FliF_C-tail_FliG_N complex (5TDY). Next, we evaluated whether contacts between FliG_N and FliF_C-tail_ observed in the crystal structures were conserved between species. Coevolved FliG_N and FliF_C-tail_ residue pairs in the concatenated coevolution matrix superimposed on the 5TDY contact map, implying that different species adopt an equivalent association between their FliF and FliG components.

The FliG_N coevolution matrix was then superimposed on the contact maps of crystal structures for FliG_N alone (*Aquifex aeolicus* PDB ID 3HJL) and in complex with FliF_C-tail_. (*T*. *maritima* 5TDY). FliG_N has a partial armadillo fold with anti-parallel α-helices that provide a characteristic signature in the contact maps for the structures. The top coevolved couplings mapped onto this feature (Fig. 4, Supplementary Information Fig. S2A). The two structures were distinguished by another anti-parallel α-helix contact that was present in the structure of FliG_N alone (Supplementary Information Fig. S2B). This contact was not supported by residue coevolution indicating that it is not part of a conserved design. *Aquifex aeolicus* and *T*. *maritima* are both thermophiles so the contact may exist only in a limited number of *A*. *aeolicus*-like species. Alternatively, it may be present in a transient assembly intermediate that has not been selected for. In any case, FliG_N seems to have evolved as part of the functional complex with FliF.

The coevolution matrix revealed a set of strongly coevolved residue pairs that did not map onto the FliG_N fold. We exploited these for construction of FliG_N dimers (Fig. 4Bii, Supplementary Information Fig. S2C). Five coevolved pairs localized at a putative dimer interface with a Cα-Cα distance of 11.0 ± 2.0 angstroms. The mean RMSD between the protomers of our dimer model and the two distinct FliG_N conformers in the crystal structure of the *T*. *maritima* FliF_C-tail_FliG_N complex were 6.3 and 6.8 angstroms (for 5TDY chains B and D respectively). The high RMSD suggests that the available crystal structures are snapshots of a conformational ensemble generated by flexure of a hinge about 30 residues upstream of the FliG_N C-terminus.

Coevolutionary information for the FliG_MC fold and inter-domain contacts was reported previously by us^39^ and others^43^. These studies documented the conservation of a “stacking contact”, formed by the apposition of three-helix armadillo motifs from each of the M and C domains. We re-examined the coevolution signal with more stringent cutoff thresholds than we used earlier to learn about dimerization of FliG_MC and, by extension, its ring assembly. We obtained a clean superposition of the coevolution matrix with the contact maps of the crystal structures with either intra-subunit (PDB ID 4FHR, Fig. 4C) or inter-subunit (“domain swap”) (PDB ID 3HJL, not shown) stacking. The coevolved pairs at the intra-subunit stack had a mean Cα-Cα distance of 15.8 ± 0.2 angstroms. This value indicated, as in the case of FliF_N.per, that the geometry of this critical interfacial contact, seen in all crystal structures thus far, is conserved. Apart from the stacking contact, the coevolution signal was too sparse for dimer model construction. Intra-subunit (4FHR) FliG_MC homodimers were obtained with rigid-body docking implying that packing interactions may exist. Residue coevolution was one line of evidence used to support domain-swap polymerization of FliG rings. Residue coevolution is certainly consistent with this idea but cannot be used to prove it.

In conclusion, there is a strong coevolution signal for a composite FliG_N.FliF_C-tail_ fold and for its dimerization, although the conformation of the FliG_N C-terminal segment that links to FliG_M differs in our model dimer from those seen in the crystal structures. These differences may be due to flexibility of this segment or broader structural variability between FliG proteins of different species. The stacking contact between FliG_M and FliG_C is also part of the conserved FliG design.

### Fits of atomic dimer models into the high-density EM sub-volumes

**Figure 5:**
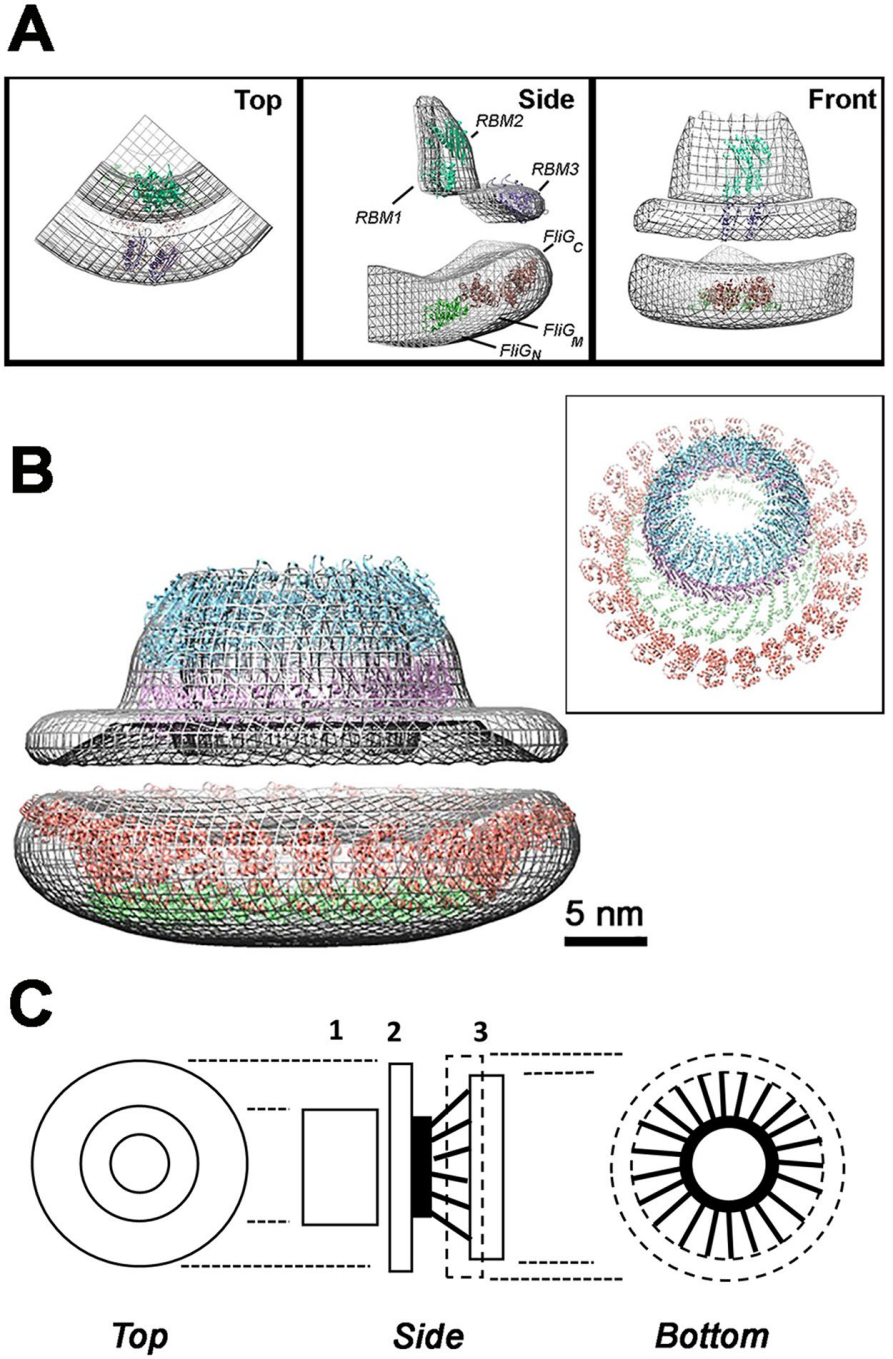
Homodimer fits of FliF and FliG domains in the 3D reconstruction suggest a mechanical model. (**A**) CHIMERA^61^ best-fits of the EscJ dimer (RBM1 + RBM2; c = 0.87), split RBM3 (c = 0.83), *T*. *maritima* FliG_N (5TDY-A, B; c = 0.81) and FliG_MC (4FHR; 0.83) into the cylindrically-averaged 3D-reconstruction (mesh representation, 0.4 contour level, c = correlation coefficient). Fits were also made for the alternative *T*. *maritima* (5TDY-D; c = 0.80) and *A*. *aeolicus* (PDB ID = 3HJL; c = 0.69) FliG_N conformations. The sub-volumes were fit sequentially; first the EscJ dimer followed by the RBM3 dimer model for the periplasmic sub-volumes; the *T*. *maritima* FliG_N followed by the FliG_MC structures for the cytoplasmic sub-volume. Manual refinement improved correlation, albeit to a small degree, of the automated assignments. (**B**) CHIMERA sequential fit of 13 dimer rings of 1YJ7, RBM3 and FliG_N plus 26 FliG_MC in the reconstruction (0.42 electron density/voxel threshold, volume = (3.81*(10^6)) cubic angstroms). **Box Inset**. Oblique view of the sequential fit ring models without the electron density map. Atomic structures in B are color coded as in A. (**C**) Engineer’s sketch of the mechanical design of the FliFFliG fusion rotor. 1 = RBM1/RBM2 ring. 2 = RBM3 ring. 3 = FliG-N (solid)/FliG-MC (dashed) rings. FliF transmembrane (black ring) and cytoplasmic C-terminal helices (black rods).

The dimer models were positioned in the high density sub-volumes of our 3D-reconstruction. The models were manually placed at approximate locations guided by proteolysis-based assignment of FliF domains to structural features^19^ and of FliG to difference analysis of pH-treated and native and fusion mutant basal bodies^24^. The manual placement was followed by automated best-fit to determine the likely orientation (Fig. 5A). The EscJ (RBM1.RBM2) dimer fit into the top ring while the RBM3 dimer model fit in the middle ring (S-ring). There is extra density in the top ring that may be filled by the predicted β-sheet hairpin. The FliG_MC dimer was positioned first in the assigned cytoplasmic sub-volume, followed by fit of the FliG_N dimer in the residual volume. The major uncertainty in the fit was the separation between the two modules. Theoretically, this separation could be greater than 5 nm based on a fully extended conformation of the α-helical FliG_N-FliG_M linker (as observed in *T*. *maritima* 5TDY chain D).

The dimer structures were then symmetrized to generate rings with 13-fold (26 subunit) symmetry consistent with the reported 26-fold symmetry for the MS-ring^38^ though the symmetry has been re-evaluated (*K*. *Namba*, *pers*. *comm*). The generated ring structures were sequentially best-fit into the high-density sub-volumes. The volume of the FliFFliG fusion (~100 kD monomer molecular weight) 26mer is 3.33*(10^6)) cubic angstroms. The fitted modules, plus the β-hairpin predicted by residue coevolution in the intervening sequence between the split RBM3 N- and C- terminal modules, constitute 78%, by sequence, of the fusion protein. The best match to the fitted volume (3.81*(10^6)) at the chosen electron density threshold (0.42) after correction for this value is a 37mer; but small shifts in threshold significantly shift the cytoplasmic volume. An alternative arrangement of FliF_C.per RBM3 juxtaposed with the EscJ ring in the top collar instead of in the S-ring illustrates the extent of the wiggle room (Fig. 5B). The S-ring could then consist of the absent β-hairpin that is at right angles to the adjacent RBM in the SpoIIIAG channel^41^. The fits have heuristic value in that they demonstrate that the dimer models have the appropriate curvature to form ring structures with diameters consistent with their positional assignments. The FliG_N ring, for example, has a diameter smaller than the FliG_MC ring, consistent with a concentric arrangement of the two rings. Accommodation of the FliG_MC rings within the high-density cytoplasmic sub-volume is consistent with a tightly-packed ring such as would be formed by the chained dimer^43,44^. The curvature is relatively insensitive to subunit symmetry. Change from 26 to 37-fold symmetry changes the tangent angle <3.5°. There are >40 FliF_C.cyt residues in addition to the C-tail that can form loose-packed, 6 nm long extended α-helices which connect the high-density cytoplasmic module to the membrane. Combined with the membrane, these would account for the low-density region between the high-density S-ring and cytoplasmic module. A higher-resolution 3D reconstruction will resolve the ambiguities considered above.

#### A residue coevolution-guided mechanical model

Eubacterial flagellar motors switch rotation direction abruptly, mostly without detectible change in rotation speed. The torque at the FliG_C/Mot rotor-stator interface must be transmitted via the MS-ring to axial basal body components, the rod, hook and filament. The FliG rotor track must make synchronous, highly co-operative changes, coupled to FliM, to re-orient and switch rotation direction^45–47^. The building blocks deduced from residue coevolution provide clues on how rotor assembly, torque transmission and synchronous switching is achieved.

Strong coevolution signals validate the folds of FliF RBMs inferred from structural homology, and also point to strong homodimer contacts. The inter-digitating loops that mediate the contacts form a tightly-packed design to promote assembly of rigid ring modules. Such modules are compatible with the high-density periplasmic sub-volumes measured in 3D EM reconstructions. Another tightly-packed module assembled through evolutionarily-conserved, homodimer contacts is the ring of co-folded FliG_N/FliF_C-tail_ domains within the cytoplasmic high-density sub-volume. Residue coevolution does not show tertiary interactions between the FliF_C. cyt segments, which are predicted to be overwhelmingly α-helical. The FliG_N sequence is contiguous with FliG_MC, but the structure of the linker between their two rings is not resolved by residue coevolution. This study re-affirms evidence for the FliG_MC stacking contact reported earlier but does not reveal dimer couplings outside such stacking contacts. The residue coevolution is in line with EM evidence, (^20,^ this study) that the FliG ring is labile, However, 3D heterogeneity classification indicates that FliG rings self-assemble to some degree in the full-frame fusion. Thus, the EM and residue coevolution argue that contacts with the distal C ring component FliM^25,26,48^ are not required for, but probably stabilize, the assembly of the FliG_MC ring.

Inspection of the coevolved contacts in the utilized structures reveals ionic, polar and hydrophobic couplings in equal measure (Supplementary Information Fig. S3A,B). There are also coevolved residue positions occupied by repulsive same charge (1) and charged-hydrophobic residue pairs (3) in these structures but have chemical compatible pairs in other species. While theories for detection of coevolved residues are well-developed, much needs to be learnt regarding the physico-chemical mechanisms underlying the coevolution. It will be important to build up the coevolved residue database to study whether couplings needed for construction of stable structural modules differ in their evolutionary trajectories from, for example, couplings for allosteric signal transmission^49,50^.

We propose a mechanical model to summarize this structural knowledge and relate it to function (Fig. 5C). The FliF RBM modules are the hub assembly in a wheel-like rotor design. They are connected via FliF transmembrane helices and FliF_C. cyt to the FliG_N module. The viscosity difference between membrane^51^ and cytoplasm^52^ aids axle alignment. The FliF_C. cyt α-helices, anchored by the transmembrane helices, are the fasteners. Their C-terminal tails (studs) are embedded in FliG_N domains (lug-nuts) in another tightly-packed ring to damp out helix bending motions^53^. The entire FliF/FliG_N assembly is designed to minimize elastic compliance during torque transmission. In contrast to the hook whose compliance facilities bundle formation^54^, compliance in internal elements will delay switching of rotation direction, particularly under high load, without compensatory benefit. Another set of α-helical connectors (spokes) whose length may be varied to tune torque, change the angle between FliG_N and FliG_MC. The FliG_MC ring, which has tensile character important for its function^55^, is the tire but with the caveat that, unlike automotive tires, it generates rather than utilizes torque. The polarity of its tread pattern and consequently the torque, switches between bi-stable states about once a second in order to optimally transduce chemotactic signals from the C-ring. A rigid, uni-stable design is not compatible with its function, as reflected in its overall evolution^39^. A high-resolution reconstruction will test and enrich the key tenets of this model.

## Methods

### Sample preparation

Plasmid pKKG7 (full frame FliFFliG fusion) was derived from pKLR1, a plasmid that co-overproduces FliF and FliG^21^, with the Qiagen Quick Change mutagenesis kit. The seven nucleotide absent in the genomic FliFFliG fusion^23^, plus an additional three nucleotides at the 3′ end were deleted with forward (5′-GATCATGAGTAATCTTAGCGGTACC-3′) and reverse (3′-CATCCACGAATGACCAG-5′) PCR primers. The strategy brought the *fliF* and *fliG* genes into frame with the FliF C-terminal MSNDHE residues replaced with I in the fusion. Plasmid pKKG1 (ΔFliFFliG fusion) also expressed well demonstrating that other segments of the full-frame fusion folded independently of the FliG_NFliF_C-tail_ attachment. The plasmids alone or in combination with plasmid pKOT179 (FliM, FliN overproducer^18^) were transformed into the *E. coli* C41 expression host (Lucigen Inc). The cells were grown, induced and lysed as reported previously^21^. FliM and FliN co-precipitated with the fusions, demonstrating that these proteins could assemble onto the fusion templates. Expression levels in the lysates, monitored by SDS-PAGE electrophoresis, were comparable to levels for the separate proteins encoded by the parent pKLR1 plasmid. Penicillin was used to partially digest the cell wall revealing inner membranes. Metal replicas of these preparations by freeze-etch electron microscopy, following reported protocols^56^, confirmed that the overproduced FliFFliG fusion ring complexes inserted into and packed the cytoplasmic membrane (Supplementary Information Fig. S1A).

After low-speed centrifugation (Sorvall RC5C super-speed centrifuge, 15,000 g, 20 minutes) to remove cell debris, the pellet from the high-speed centrifugation (Beckman Optima ultracentrifuge, 100,000 g, 1 hour) was resuspended in 2 ml buffer C (10 mM Tris, 1 mM EDTA, pH 8.0), re-centrifuged, resuspended in pH 11.0 buffer to dissolve outer membrane vesicles. The final pellet, resuspended in 400 μl pH 8.0 buffer C, was layered onto a pre-formed 10–50% sucrose density gradient. After centrifugation (Beckman TL100 ultracentrifuge, 2 hours, 40,000 g), the band containing the complexes was collected and the sucrose dialyzed out prior to grid preparation. 4.8 µl of protein solution was applied to 200 mesh Cu grids (Electron Microscopy Sciences) coated with a continuous carbon film and then incubated at room temperature for 30 seconds with the excess solution blotted away. The grid was then washed twice in buffer and stained with a 0.7% uranyl formate four times, with excess blotted away each time. On the fourth addition of uranyl formate, the stain was incubated for 30 seconds, then blotted to dryness. Grids were imaged on a Tecnai T12 (FEI) operating at 120 kV and equipped with a Gatan 4 K × 4 K camera. 2400 micrographs were collected by the EPU automatic data acquisition software (FEI) at a nominal magnification of 50,000× and a pixel size of 2.09 Å/pixel at low dose (~15–20 electrons/square angstrom). The defocus range was 5–6 μm (Supplementary Information Fig. S1A).

### Image reconstruction

The contrast transfer function (CTF) was estimated on all micrographs by CTFFIND3^57^, with further image processing conducted in RELION 1.4^58^. About 650 particles were initially picked manually; 2D class averages from these were used as templates for automated picking. 98,000 automatically picked particles were extracted for 2D classification. 15 rounds of 2D classification (Supplementary Information Fig. S1B) plus 5 rounds of 3D classification and refinement resulted in a final set of 27,450 particles, distributed over 5 3D classes (Supplementary Information Fig. S1C). We pooled particles from 3D Classes 2 and 4, 43% of the total population, that had more extensive cytoplasmic structure than 3D classes 1,3 and 5. Two 3D classification cycles and refinement of the pooled population yielded a dominant (77%) 3D class that was used as the final map. Local resolution was estimated with RESMAP^59^. The maps were displayed and their parameters (electron density, volume, diameter) were computed in UCSF CHIMERA^60,61^.

### Residue coevolution

Coevolution analysis used a custom version^39^ of PSiCOV^11^, but with a more stringent cut-off (>3σ rather than >2σ) threshold than previously. The lower (2σ) threshold included couplings that we have reported as associated with long-range allosteric communication^55^ or conformational transitions^62^. Coupling strength between residue positions (*I*,*j*) is reported as a PSICOV score $P{S}_{ij}$ calculated for the 20 × 20 inverse covariance submatrix ($\varnothing )\,$for the twenty amino acid types ($a,b$) at each position^11^.

1 $$P{S}_{ij}=\,\sum _{ab}|{\varnothing }_{ij}^{ab}|$$

Scores were reported as normalized scores^11^ after average product correction to account for entropic and phylogenetic bias^63^. A typical tailed score distribution is shown (Supplementary Material Fig. S3C). Randomization of the coevolution matrix resulted in a symmetric distribution with loss of coevolution signal^39^. Studies have shown that the ratio of MSA sequence depth (number, $N$) to the protein residue length $L$ should be >0.3 for retrieval of statistically meaningful coevolution information^13^.

Results were double-checked and composite FliF.FliG sequences concatenated and analyzed with GREMLIN^64^. MSAs for FliF_N.per (PF01514), FliF_C.per (PF08345), FliG_N (PF14842), FliG_M (PF14841) and FliG_C (PF01706) were retrieved from the Pfam database. Sequences in the MSAs were clustered with CD-Hit^65^, then filtered to remove non-flagellar sequence clusters from the MSAs for further analysis. X-ray structures 1YJ7.pdb (*E*. *coli* EscJ), 5TDY.pdb (*T*. *maritima* FliF_C-tail_, FliG_N), 4FHR.pdb (*T*. *maritima* FliM_M.FliG_MC) and 3HJL.pdb (*A*. *aeolicus* FliG) were downloaded from Protein Data Bank. The RBM3 model was downloaded from^36^ Supplemental Files. The X-ray structure sequences were added to the PFAM alignments with MAFFT^66^. Residue positions absent in the structure sequences were removed from the alignments. HMMER3^67^ and MUSCLE^68^ were used for *de novo* MSA construction. TMHMM^69^ and PsiPred^70^ were used for transmembrane helix and secondary structure prediction respectively.

### Coevolution-guided dimer models

The top coevolved residue pairs, filtered by opposing interfacial location and explicitly restrained to be within 10 Å Cα-Cα distance, were included as constraints in all model calculations. The protein threading server I-Tasser^71^ was used to model a fold for the intervening sequence between the split RBM3 segments. Sequence homology was examined with the EMBL-EBI local pairwise alignment server^72^. EscJ and chained FliG-MC dimers were downloaded from the 1YJ7.pdb and 3HJL.pdb structures respectively. The Haddock 2.2 server^73^ was used to model dimers for the split RBM3, FliG_N and FliG_MC from the monomer structures with flexible-fit protocols. Eight coevolved pairs (plus another eight to validate the split monomer fold) were used for construction of the split RBM3 dimer. Coevolved surface-exposed residues located on opposing sides of the monomer with high scores (>0.2) were input as constraints. Representatives of the best-scoring RBM3 clusters with dimerization modes similar to at least one of the published RBM oligomer crystal structures were redocked in Haddock with the coevolved pairs. The top structure in the cluster with the best score was selected. Five coevolved residue pairs with partners on opposing surfaces were selected for construction of the FliG-N dimer. Representative structures of the best-scoring clusters selected as initial models were rebuilt with COOT^74^ to equalize monomer conformations and minimize clashes between them or with their associated FliF tails. Finally, backbone geometry and side-chain contacts were optimized with FG-MD^75^. The results were compared against ZDock^76^ rigid-body docking of dimer models with the root mean square deviation (RMSD) as a metric. Dimer models for intra-stack FliG_MC were generated by ZDock but lacked support from residue coevolution.

### Data availability statement

The datasets generated during and/or analyzed during this study are available from the corresponding author on reasonable request.

## Electronic supplementary material

Supplementary Information
