# Supplementary material for: A coevolution-guided model for the rotor of the bacterial flagellar motor
Source: Sci Rep. 2018 Aug 6;8:11754. doi: 10.1038/s41598-018-30293-0 (PMC6079021; doi:10.1038/s41598-018-30293-0)
Supplement: Supplementary file 1 — Supplementary Information [file 41598_2018_30293_MOESM1_ESM.pdf]

SUPPLEMENTARY INFORMATION

A coevolution-guided model for the rotor of the bacterial flagellar motor

Shahid Khan[1,2], Tai Wei Guo[1] & Saurav Misra[1,3]

[1]Laboratory of Cell Biology, Center for Cancer Research, National Cancer Institute, NIH, Bethesda, MD 20892
[2]Molecular Biology Consortium, Lawrence Berkeley National Laboratory. Berkeley, CA 94720.
[3]Current Address: Department of Biochemistry & Molecular Biophysics, Kansas State University, Manhattan, KS 66506

Biochemistry. Microscopy & Image Reconstruction

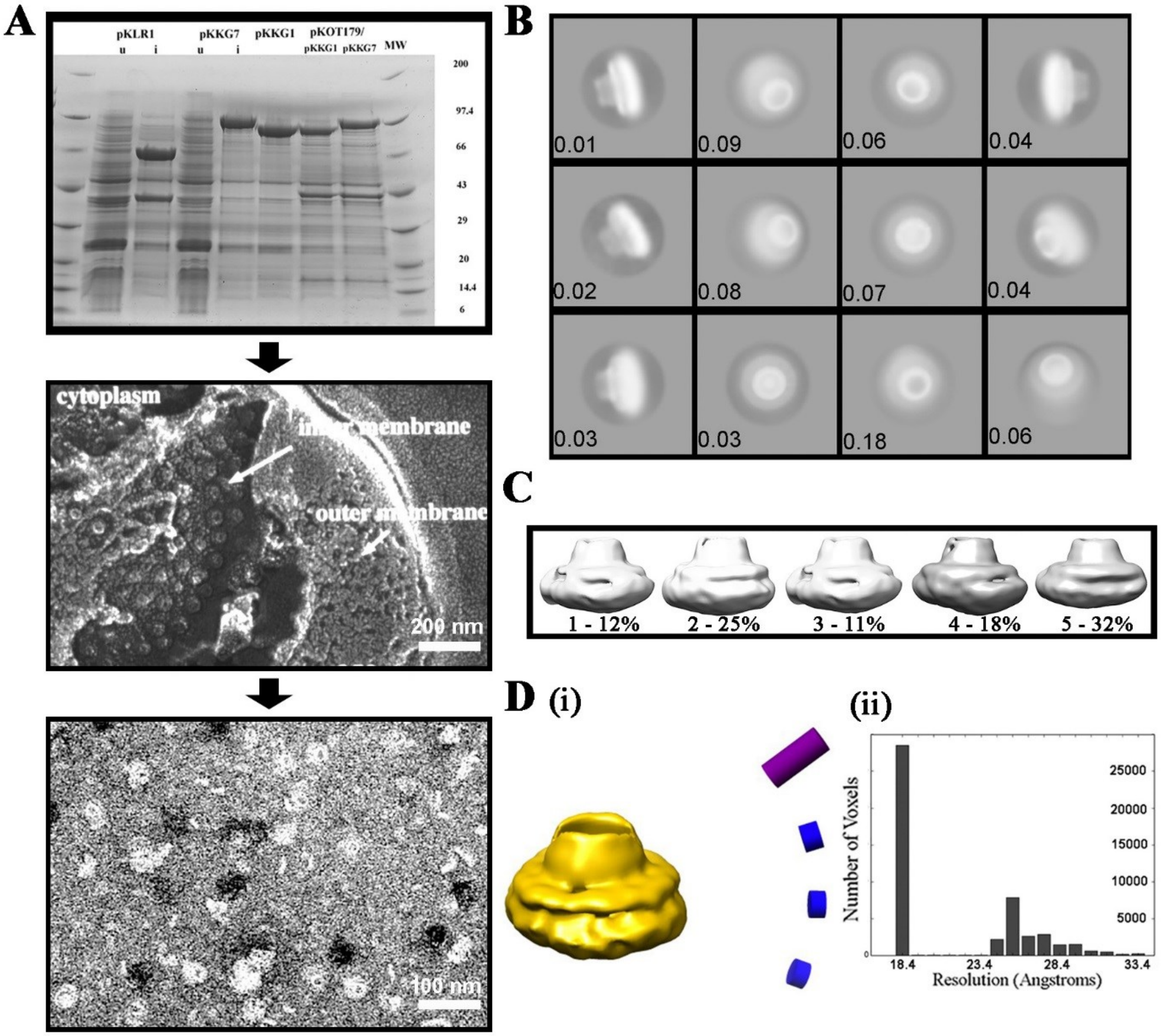

Figure S1: Steps in the 3D EM reconstruction. A. Stages in sample preparation. **Top** - Protein expression checked by SDS-Page gel electrophoresis. **Middle** – FliF.FliG fusion complex assembly and insertion into cytoplasmic membrane checked by freeze-etch electron microscopy. **Bottom** – Isolated complexes visualized by negative-stain electron microscopy. **B.** 2D-classes after 15 rounds. Numbers indicate population fraction. **C.** 3D-classes after 5 rounds. Population fractions (%) are indicated. Population size = 27,450. **D** (**i**). Orientation distribution of particles used for the final 3D-reconstruction. Rod length and color (blue (low) to red (high)) indicate fractional contribution of population in the orientation indicated

by the rod. (**ii).** Histogram of voxel resolution. Voxels at 18.4% resolution constitute over half of the total population.

The Coevolution Signal – Conformation Discrimination & Biochemical Rationale

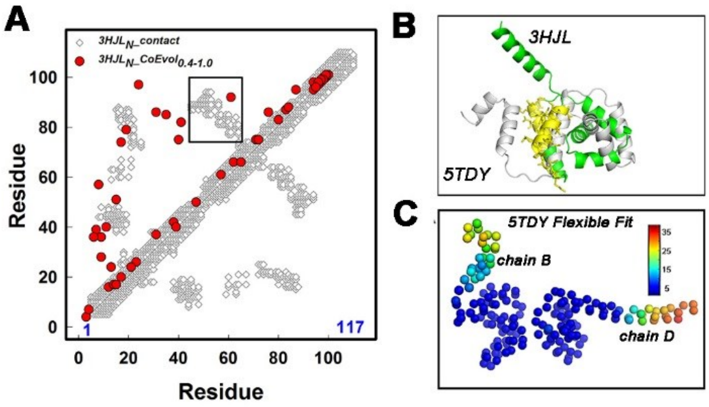

Figure S2: Supplemental analysis of the FliG-N coevolution signal. (A) The FliG-N coevolution matrix (red) superimposed on the contact map for 3HJL. The is no coevolution signal for the contact between 3HJL helices 4 and 5 (box). 5TDY.pdb = *T.maritima* $FliF_{C\text{-}tail}$FliG_N complex. 3HJL.pdb = *A. aeolicus* FliG. **(B)** Alignment of FliG-N structures. Superimposed 3HJL (green / yellow) and 5TDY (white) crystal structures (RMSD = 1.09 angstroms). The interaction between 3HJL helices 4 and 5 (yellow) is absent in 5TDY. **(C)** The alteration of the initial structure (5TDY-BB) during flexible fit to obtain the FliG-N dimer is recorded by the root mean square deviation (RMSD) of the residues after structural alignment of the initial and final model. Vertical bar shows color-coded $(RMSD)^2$ range ($angstrom^2$).

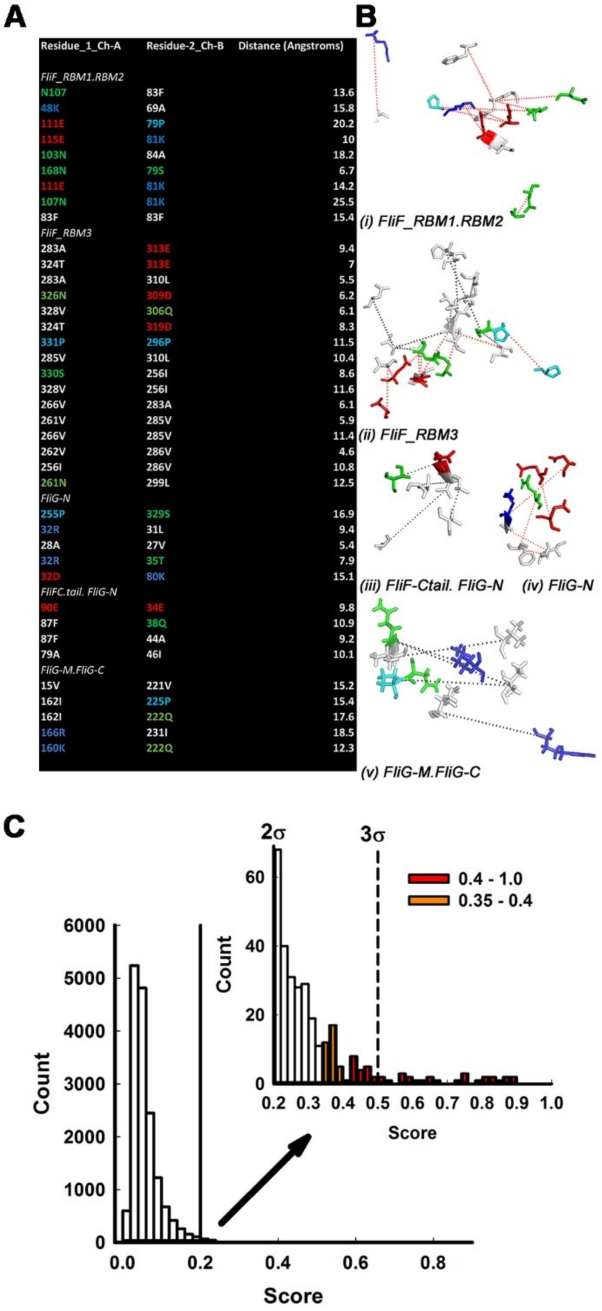

Figure S3: Analysis and Selection of Coevolved Contacts: 5TDY.pdb = *T.maritima* FliF$_{C\text{-}tail}$FliG_N complex. 4FHR.pdb = *T. maritima* FliG_MC. 3HJL.pdb = *A. aeolicus* FliG. Biochemistry of coevolved inter-domain contacts. **(A)** Identities and Cα distances of residues at interfacial positions in the crystal structures used for interpretation of the coevolution matrix. **(B)** The 3D network of coevolved residue position at the interfacial contacts examined in this study. **(C)** Normalized PSICOV score distribution histogram for 4FHR FliG_MC shown relation of selected coevolved pairs for contact detection (colored bars) to the overall distribution.
